# Simultaneous spatial and temporal regularization in low‐dose dynamic contrast‐enhanced CT cerebral perfusion studies

**DOI:** 10.1002/acm2.13983

**Published:** 2023-04-06

**Authors:** Kenya Murase, Atsushi Nakamoto, Noriyuki Tomiyama

**Affiliations:** ^1^ Department of Future Diagnostic Radiology Graduate School of Medicine Osaka University Suita Osaka Japan; ^2^ Department of Diagnostic and Interventional Radiology Graduate School of Medicine Osaka University Suita Osaka Japan

**Keywords:** cerebral perfusion study, low‐dose dynamic contrast‐enhanced CT, low‐rank and sparse decomposition, simultaneous spatial and temporal regularization, total generalized variation, total variation

## Abstract

**Purpose:**

To apply total generalized variation (TGV) and its combination with low‐rank and sparse decomposition (LRSD) (LTGV) to cerebral perfusion studies using low‐dose dynamic contrast‐enhanced (DCE) CT and to quantitatively evaluate their performances through comparisons with those without any regularizers and those of total variation (TV) and its combination with LRSD (LTV) using simulation and clinical data.

**Methods:**

The simulation study used a realistic digital brain phantom. Low‐dose DCE‐CT images were reconstructed using the regularizers and primal‐dual algorithm. Subsequently, cerebral perfusion parameter (CPP) images were generated from them. Thereafter, their quality was evaluated based on the peak signal‐to‐noise ratio (PSNR) and structural similarity index measure (SSIM). Further, the accuracy of CPP estimation was evaluated through a linear regression analysis between the CPP values obtained by the above regularizers and those obtained from the noise‐free DCE‐CT images. In addition, the mean and standard deviation of the CPP were calculated (region analysis). In the clinical study, low‐dose DCE‐CT images were generated using normal‐dose images acquired from a patient, and CPP images were generated from them similar to that in the simulation study.

**Results:**

When using LTV and LTGV, both PSNR and SSIM were higher than those of the other methods with increasing regularization parameter values. The results of the linear regression and region analyses demonstrated that TGV generally exhibited the best performance, followed by LTGV, and finally that of TV was significantly different from those of the other regularizers. Despite an overall consistency between the simulation and clinical results, certain inconsistencies appeared owing to the difference in generating low‐dose DCE‐CT images.

**Conclusions:**

The results implied that TGV and LTGV were useful in improving the accuracy of CPP estimation using low‐dose DCE‐CT. This study provides an improved understanding of the performance of regularizers and is expected to aid in the selection of a suitable regularizer for low‐dose DCE‐CT perfusion studies.

## 1 INTRODUCTION

Dynamic contrast‐enhanced computed tomography (DCE‐CT) is a promising tool for analyzing hemodynamic changes in tissues and organs.^1^ It enables the conduction of cerebral perfusion studies and the quantification of cerebral perfusion parameters, such as cerebral blood flow (CBF), cerebral blood volume (CBV), and mean transit time (MTT). Assessing the state of perfusion in the brain using these perfusion parameters is important for determining medical treatment plans and/or predicting prognosis in patients with cerebrovascular diseases.^2^

Radiation exposure during DCE‐CT perfusion studies is a serious problem that is preventing its widespread employment.^3^ Reducing the x‐ray tube current of CT is one solution for reducing the radiation dose to patients. However, this increases the statistical nose in CT images.^4^ Thus, various methods including filtering techniques, such as Gaussian filtering^5^ and time‐intensity profile similarity bilateral filtering,^6^ and partial differential equation‐based methods, such as an anisotropic diffusion method,^7^ have been proposed to reduce the noise in CT images.

Recently, reflecting the age of artificial intelligence (AI), there have been attempts made to use convolutional neural networks (CNNs) for denoising medical images.^8^, ^9^, ^10^, ^11^ To facilitate the application of these methods to CT images, CNNs are trained to map low‐dose CT images to normal‐ or high‐dose CT images using a training set with paired or unpaired low‐ and normal‐ or high‐dose CT images.^9^, ^10^, ^11^ The use of a deep image prior^12^ and self‐supervised approach^13^ have also been proposed. Despite these approaches appearing promising, further studies may be necessary to establish their usefulness in the clinical setting.

With the emergence of compressed sensing (CS),^14^ sparsity‐inducing regularizers, such as the total variation (TV) norm have been used as powerful tools for enforcing piecewise smoothness in image processing.^15^ These approaches have also been applied to DCE‐CT perfusion studies for denoising their images.^16^, ^17^, ^18^ In the field of magnetic resonance imaging (MRI), the theory of CS has proven the possibility of accurate reconstruction of dynamic MRI images from under sampled k‐space data under specific constraints. This demonstrates that CS is a promising method for accelerating dynamic MRI without reducing the spatial and temporal resolution.^14^, ^19^ In addition to the TV,^20^ the uses of total generalized variation (TGV),^21^ low‐rank and sparse decomposition (LRSD),^22^ nuclear norm (NN) minimization,^23^ and their combinations have been proposed to provide constraints such as the sparsity and piecewise smoothness of the reconstructed images as regularizers.^24^, ^25^, ^26^, ^27^ However, to the best of our knowledge, the TGV and its combination with LRSD have not been applied to DCE‐CT perfusion studied and their performances are yet to be investigated.

When solving optimization problems in image denoising and reconstruction, the selection of hyperparameter (regularization parameter) values is not a straightforward task. In general, this is performed through trial and error using hand tuning. When the number of regularizers, and hence the number of hyperparameters to be optimized, are significant and/or the problem is ill‐posed, considerable time and effort are required to achieve their optimal values, particularly in case of random searches. If a parameter that can be used as a metric for reducing the search range and/or the performance of hyperparameters is known, the search time and effort are reduced. In practical applications, a method that is insensitive and flexible to the choice of hyperparameter values is desirable. Therefore, the performance of regularizers when varying their regularization parameter values must be investigated in detail.

This study aimed to apply TGV and its combination with LRSD to low‐dose DCE‐CT cerebral perfusion studies and to quantitatively investigate their performances in comparison with those without any regularizers and those of the conventional TV and its combination with LRSD, using simulation and clinical data.

## 2 MATERIALS AND METHODS

### 2.1 Image reconstruction

#### 2.1.1 Image reconstruction using filtered back‐projection method

In this study, we used the filtered back‐projection (FBP) method to perform comparisons. First, DCE‐CT images at each slice and each frame were reconstructed from noise‐free projection data (sinogram) using the FBP method with a Ram–Lak filter^28^ for equiangular fan‐beam scanning geometry.^29^ These images were used as a reference for the comparison of performances of different methods under the same scanning geometry. In this study, these images are referred to as “REF”.

The DCE‐CT images were also reconstructed from noisy projection data using the FBP method with Ram–Lak^28^ and Shepp–Logan filters.^30^ These methods are referred to as “FBP(RL)” and “FBP(SL)”, respectively.

#### 2.1.2 Image reconstruction using TV

The image reconstruction of DCE‐CT images using TV is formulated as follows:

(1) $$\;\hat{x}=\underset{x}{\mathrm{argmin}}\;\frac{1}{2}∥Ax-b{∥}_{F}^{2}+\alpha ∥\nabla_{3}x{∥}_{1},$$

where ***x*** denotes the DCE‐CT image matrix comprising $x_{i}\;\left(i\;=\;1,2,\cdots ,N_{f}\right)$, with ***x***_*i*_ and *N_f_* being the image matrix at temporal frame *i* and the total number of frames, respectively, ***b*** is the projection data matrix, ***A*** denotes the sampling operator (the system matrix), $∥\cdot {∥}_{F}$ and $∥\cdot {∥}_{1}$ denote Frobenius and ℓ_1_ norms, respectively, ∇_3_ is the three‐dimensional gradient operator comprising spatial and temporal gradient operators,^27^ that is, $\nabla_{3}=\left(\nabla_{x},\;\nabla_{y},\nabla_{t}\right)\;$ with $\nabla_{x}$, $\nabla_{y}$, and $\nabla_{t}$ being the gradient operator along the *x*, *y*, and temporal directions, respectively, the symbol *α* is a regularization parameter for balancing the data consistency and sparsity, and $\hat{x}$ denotes the reconstructed image matrix. Equation (1) implies that the DCE‐CT images are reconstructed such that the sum of the Frobenius norm between the projection data calculated from the images (***A*** ***x***) and measured projection data (***b***) and the ℓ_1_ norm of the spatial and temporal gradients of the images ($∥\nabla_{3}x{∥}_{1}$) is minimized. In this study, this method is referred to as “TV”.

#### 2.1.3 Image reconstruction using TGV

The image reconstruction of the DCE‐CT images using a second‐order TGV is formulated as follows:

(2) $$\;\hat{x}=\underset{x}{\mathrm{argmin}}\;\frac{1}{2}∥Ax-b{∥}_{F}^{2}+TGV_{\alpha }^{2}\left(x\right),$$

where $TGV_{\alpha }^{2}$ denotes the second‐order TGV and is defined as follows^31^:

(3) $$TGV_{\alpha }^{2}\left(x\right)=\min_{v}\;\alpha_{1}∥\nabla_{3}x-v{∥}_{1}+\alpha_{0}∥E\left(v\right){∥}_{1},$$

where α_0_ and α_1_ denote the regularization parameters. In Equation (3), E(v) denotes the symmetric gradient of ***v*** and is expressed as $E\left(v\right)=\left(\nabla_{3}v+\nabla_{3}v^{T}\right)/2\;$, where *T* denotes the transpose of a matrix. The first term on the right‐hand side of Equation (3) is for effective preservation of the edge information of the reconstructed image. Further, the second term implies effective improvement of the smoothness of the image because the second‐order derivative of the image ($\nabla_{3}^{2}x$) is small within the smooth region of the image.^31^ In this study, this method is referred to as “TGV”.

#### 2.1.4 Image reconstruction using combination of LRSD and TV

When image reconstruction of DCE‐CT images is performed using a combination of LRSD and TV, ***x*** is decomposed into low‐rank (L) and sparse (S) components, and these components are obtained by solving the following minimization problem:

(4) $$\min_{L,\;S}\;\frac{1}{2}∥A\left(L+S\right)-b{∥}_{F}^{2}+\alpha ∥\nabla_{3}S{∥}_{1}+\beta ∥L{∥}_{*},$$

where ***L*** and ***S*** are the L and S components of ***x***, respectively, and $∥L{∥}_{*}$ denotes the NN (Schatten *p*‐norm with *p* = 1) of ***L***, that is, the sum of the singular values in ***L***. Further, the symbol β denotes the regularization parameter. In this method, $\hat{x}$ is calculated using $\hat{x}=\hat{L}\;+\hat{S}$, where $\hat{L}$ and $\hat{S}$ denote the L and S components obtained by solving Equation (4), respectively. In this study, this method is referred to as “LTV”.

#### 2.1.5 Image reconstruction using combination of LRSD and TGV

As in LTV, when using a combination of LRSD and second‐order TGV, ***x*** is decomposed into L and S components, and these components are obtained by solving the following minimization problem^27^:

(5) $$\min_{L,\;S}\;\frac{1}{2}∥A\left(L+S\right)-b{∥}_{F}^{2}+TGV_{\alpha }^{2}\left(S\right)+\beta ∥L{∥}_{*},$$

and $\hat{x}$ is calculated as $\hat{x}=\hat{L}\;+\hat{S}$, where $\hat{L}$ and $\hat{S}$ denote the L and S components obtained by solving Equation (5), respectively. In this study, this method is referred to as “LTGV”.

#### 2.1.6 Execution of image reconstruction

The primal‐dual (PD) algorithm^32^, ^33^ was used to estimate $\hat{x}$ by solving Equations (1)–(5). The details of the PD algorithm are described in the Appendix. The iterative procedure of the PD algorithm was repeated until $∥{\hat{x}}^{n+1}-{\hat{x}}^{n}{∥}_{F}/∥{\hat{x}}^{n}{∥}_{F}<\varepsilon_{\mathrm{tol}}$ was satisfied, or the maximum number of iterations ($N_{max}$) was reached, where ${\hat{x}}^{n}$ denotes the reconstructed image matrix at iteration *n*. In this study, $\varepsilon_{tol}$ and $N_{max}$ were set to 10^−6^ and 500, respectively. When using TV and TGV, the initial estimate (${\hat{x}}^{1}$) was given by the image matrix reconstructed using the FBP(RL). When using LTV and LTGV, the L component of ${\hat{x}}^{1}$ was also given by the image matrix reconstructed using FBP(RL), whereas the S component was set to a zero matrix.

The regularization parameters *α* and *α*_1_ were varied between 0.0001 and 0.01, whereas *α*_0_ was set as double of *α*_1_ (recommended by Knoll et al.^34^) and *β* was set to 2.0. When using the PD algorithm (see Appendix), both the first‐ (*σ*) and second‐order step sizes (*τ*) were set to 0.25. These values were determined through trial and error using hand tuning with reference to previous studies.^27^, ^35^ These values were used in both simulation and clinical studies.

### 2.2 Generation of cerebral perfusion parameter images

According to the indicator dilution theory for intravascular contrast agents (CA), the time‐dependent concentration of CA in the volume of interest (VOI) ($C_{\mathrm{VOI}}\left(t\right)$) is expressed as^36^

(6) $$C_{\mathrm{VOI}}\left(t\right)=\frac{\rho }{k_{H}}\mathrm{CBF}\int_{0}^{t}C_{\mathrm{AIF}}\left(\tau \right)\cdot R\left(t-\tau \right)d\tau .$$

In Equation (6), $C_{AIF}\left(t\right)$ is the time‐dependent concentration of the CA in a feeding artery, that is, arterial input function (AIF). R(t) is the residue function. This function is the relative amount of CA in the VOI in an idealized perfusion experiment, where a unit area bolus was instantaneously injected (R(0)=1) and subsequently washed out by perfusion (R(∞)=0). The symbols *ρ* and *k*_H_ denote the density of brain tissue (1.04 g/mL) and the correction factor for considering the difference in the hematocrit between large and small vessels (0.73), respectively.^36^ As per Equation (6), the initial height of R(t) equals the CBF multiplied by $\rho /k_{H}$. In this study, we used an algebraic approach based on block‐circulant singular value decomposition^37^ to calculate R(t) using Equation (6) by deconvolution, and subsequently obtained the CBF value from the maximum value of R(t). Thus, CBF images were generated though the pixel‐by‐pixel application of this approach.

CBV is expressed as

(7) $$\mathrm{CBV}=\frac{k_{H}}{\rho }\frac{\int_{0}^{\infty }C_{\mathrm{VOI}}\left(\tau \right)d\tau }{\int_{0}^{\infty }C_{\mathrm{AIF}}\left(\tau \right)d\tau }.$$

From the central volume principle, MTT is expressed as

(8) $$\mathrm{MTT}=\frac{\mathrm{CBV}}{\mathrm{CBF}}\;.$$

The CBV and MTT images were generated via the application of Equations (7) and (8) pixel by pixel, respectively. The units of CBF, CBV, and MTT were mL/100 g/min, mL/100 g, and s, respectively.

Following the administration of CA, the increase in the linear attenuation coefficient (*μ*) of the tissue was proportional to the concentration of the CA, and the proportionality constant between them was dependent on the energy spectrum of the X ray passing through the tissue and atomic constituents of the CA.^38^ When the beam‐hardening effect is negligible or properly corrected, the proportionality constant not varying among different tissues is considered a good approximation.^38^ Therefore, it can be assumed that the concentration of the administered CA is proportional to an increase in CT number, that is, contrast enhancement (CE), and $C_{VOI}\left(t\right)$ and $C_{AIF}\left(t\right)$ in Equation (6) can be replaced by the CE in the VOI and feeding artery, respectively.

### 2.3 Simulation study

#### 2.3.1 Generation of DCE‐CT and cerebral perfusion parameter images

**FIGURE 1:**
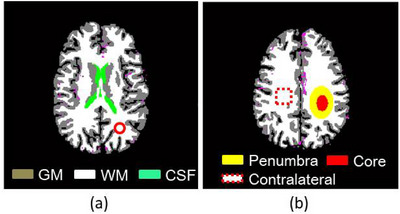
Illustration of the brain (a) without any lesions at the level of the caudate nucleus (slice #140) in the digital brain phantom^39^ and (b) with an ischemic lesion at the level of the centrum ovale (slice #152). Gray, white, and green regions illustrate the gray matter (GM), white matter (WM), and cerebrospinal fluid (CSF), respectively. Yellow and red regions illustrate the penumbra and ischemic core, respectively, and the square surrounded by a red dotted line illustrates the region of interest drawn on the contralateral normal region.

In this study, a realistic digital brain phantom with skull and blood vessels, developed by Aichert et al.^39^ was used. The DCE‐CT images for each slice were generated to comprise 256 × 256 pixels in size and 50 frames with a sampling time of 1 s. Figure 1a illustrates the brain without any lesions at the level of the caudate nucleus (slice #140 in the digital brain phantom^39^). Figure 1b illustrates the brain with an ischemic lesion at the level of the centrum ovale (slice #152). In Figure 1b, yellow and red regions illustrate the penumbra and ischemic core, respectively, and the region surrounded by a red dotted square (18 × 18 pixels in size) illustrates the region of interest (ROI) drawn on the contralateral normal region.

Poisson noise was added to the projection data to simulate statistical noise. The projection data were generated from the *μ* maps transformed from the digital brain phantom (Figure 1) using a simple monoenergetic forward model under the assumption that the *μ* values for air (−1000 Hounsfield Unit (HU)) and water (0 HU) were 0 and 0.239 cm^−1^, respectively. The *μ* value for water was obtained from the photon cross sections database provided by XCOM (https://physics.nist.gov/PhysRefData/Xcom/) for an effective x‐ray energy of 80 kVp (46 keV).^40^ We considered a fan‐beam projection geometry with 1000 projections equally spaced over a 360° angular range. The x‐ray detector was assumed to be arranged in an arc shape with 377 sensors. The source‐to‐isocenter and isocenter‐to‐detector distances were 570 and 470 mm, respectively. Further, the system matrix (***A*** in Equation 1) was computed using the standard Siddon's algorithm.^41^ The incident x‐ray photons were assumed to pass through an object according to the Beer–Lambert law; that is, the number of surviving photons (*I*) was estimated from $I\;=I_{0}\;e^{-P}$, where *I*_0_ and *P* denote the number of incident photons and the line integral of the *μ* values along the projection ray passing through the object, respectively. Unless specifically stated, *I*_0_ was assumed to be 2.5 × 10^5^ for each ray. Furthermore, readout noise obeying a Gaussian distribution with a variance of 10 was added. As the number of surviving photons reached zero or became negative, it was clamped to unity to avoid the logarithm of zero and negative values. According to Zeng et al.,^42^ *I*_0_ value of 2.5 × 10^5^ corresponds to an exposure of approximately 66 mAs.

CE images were generated through calculations of the average of each pixel intensity of the DCE‐CT images between the first and 8th frames (baseline) and subtracting it from each pixel intensity. These CE images were used to generate CBF, CBV, and MTT images using Equations (6)–(8).

The AIF ($C_{AIF}\left(t\right)$ in Equation 6) was obtained by manually drawing a square ROI (20 × 20 pixels) surrounding the internal carotid artery (ICA) in the slice including the ICA (slice #93 in the digital brain phantom^39^) and applying fuzzy c‐means clustering^43^ to this ROI.

#### 2.3.2 Evaluation of DCE‐CT images

Reconstructed DCE‐CT images were quantitatively evaluated using two measures. The first measure was the peak signal‐to‐noise ratio (PSNR), defined as

(9) $$\mathrm{PSNR}=-10\cdot \mathrm{lo}g_{10}\frac{∥{\hat{x}}_{\mathrm{dce}}-{\hat{x}}_{\mathrm{ref}}{∥}_{F}^{2}/N_{\mathrm{tot}}}{\max{\left({\hat{x}}_{\mathrm{ref}}\right)}^{2}},$$

where ${\hat{x}}_{\mathrm{dce}}$ and ${\hat{x}}_{\mathrm{ref}}$ denote the DCE‐CT image matrix reconstructed using different methods and REF image matrix, respectively, and *N* *_tot_* denotes the total number of pixels.

The other measure was the structural similarity index measure (SSIM) proposed by Wang et al.^44^ The SSIM at each frame was calculated as follows:

(10) $$\mathrm{SSIM}=\frac{\left(2\mu_{\mathrm{dce}}\mu_{\mathrm{ref}}+C_{1}\right)\left(2\sigma_{\mathrm{cov}}+C_{2}\right)}{\left(\mu_{\mathrm{dce}}^{2}+\mu_{\mathrm{ref}}^{2}+C_{1}\right)\left(\sigma_{\mathrm{dce}}^{2}+\sigma_{\mathrm{ref}}^{2}+C_{2}\right)}\;,$$

where $\mu_{dce}$ and $\mu_{ref}$ denote the averages within the window set on the DCE‐CT image at each frame reconstructed using different methods and the corresponding REF image, respectively, $\sigma_{dce}^{2}$ and $\sigma_{ref}^{2}$ denote the variances within the window set on the reconstructed DCE‐CT and REF images, respectively, $\sigma_{cov}$ denotes the covariance between the window set on the reconstructed DCE‐CT and REF images, and *C*_1_ and *C*_2_ denote two variables that stabilize the division with a small denominator. In this study, the SSIM for each frame was calculated using Equation (10) with the window size, *C*_1_, and *C*_2_ set to the default values reported by Wang et al.^44^ In addition, the average for all frames was used to evaluate the overall structural similarity between the reconstructed DCE‐CT and REF images.

#### 2.3.3 Linear regression and ROI analyses of cerebral perfusion parameters

Linear regression analysis was performed to quantitatively compare CBF, CBV, and MTT images obtained from DCE‐CT images reconstructed using different methods in cases without any lesions (Figure 1a). For CBF, the correlation coefficient (CC) between the CBF values within the brain obtained from REF (x‐axis) and those obtained from the DCE‐CT images reconstructed using different methods (y‐axis), as well as the slope and y‐intercept of the regression equation between them were calculated. These calculations were performed for CBV and MTT assays as well.

In addition, ROI analysis was performed to calculate the mean and standard deviation (SD) of CBF, CBV, and MTT in the penumbra, ischemic core, and contralateral normal regions in the case of an ischemic lesion (Figure 1b).

### 2.4 Clinical study

In this study, we used DCE‐CT images acquired from a 70‐year‐old male patient with occlusive cerebrovascular disease.^45^ After obtaining informed consent from the patient, iodinated CA (30 mL) was injected at a rate of 4 mL/s into an antecubital vein with a power injector, and continuous scans (1 s per rotation × 60) of four 5‐mm‐thick contiguous slices were performed using a multi‐detector‐row CT scanner (LightSpeed QX/i, GE Yokogawa Medical Systems, Tokyo, Japan) with an x‐ray tube voltage of 80 kVp and a tube current of 200 mA (normal dose).

To avoid scanning a patient twice, we generated the projection data of low‐dose DCE‐CT images from normal‐dose DCE‐CT images using the method described in Section 2.3.1. Similar to that in the simulation study, low‐dose DCE‐CT images were reconstructed from these projection data using FBP(RL), FBP(SL), TV, TGV, LTV, and LTGV. Subsequently, the CBF, CBV, and MTT images were generated from the CE images calculated from the reconstructed DCE‐CT images. In this study, the images generated from the normal‐dose DCE‐CT images reconstructed using FBP(RL) are referred to as “REF”.

## 3 RESULTS

**FIGURE 2:**
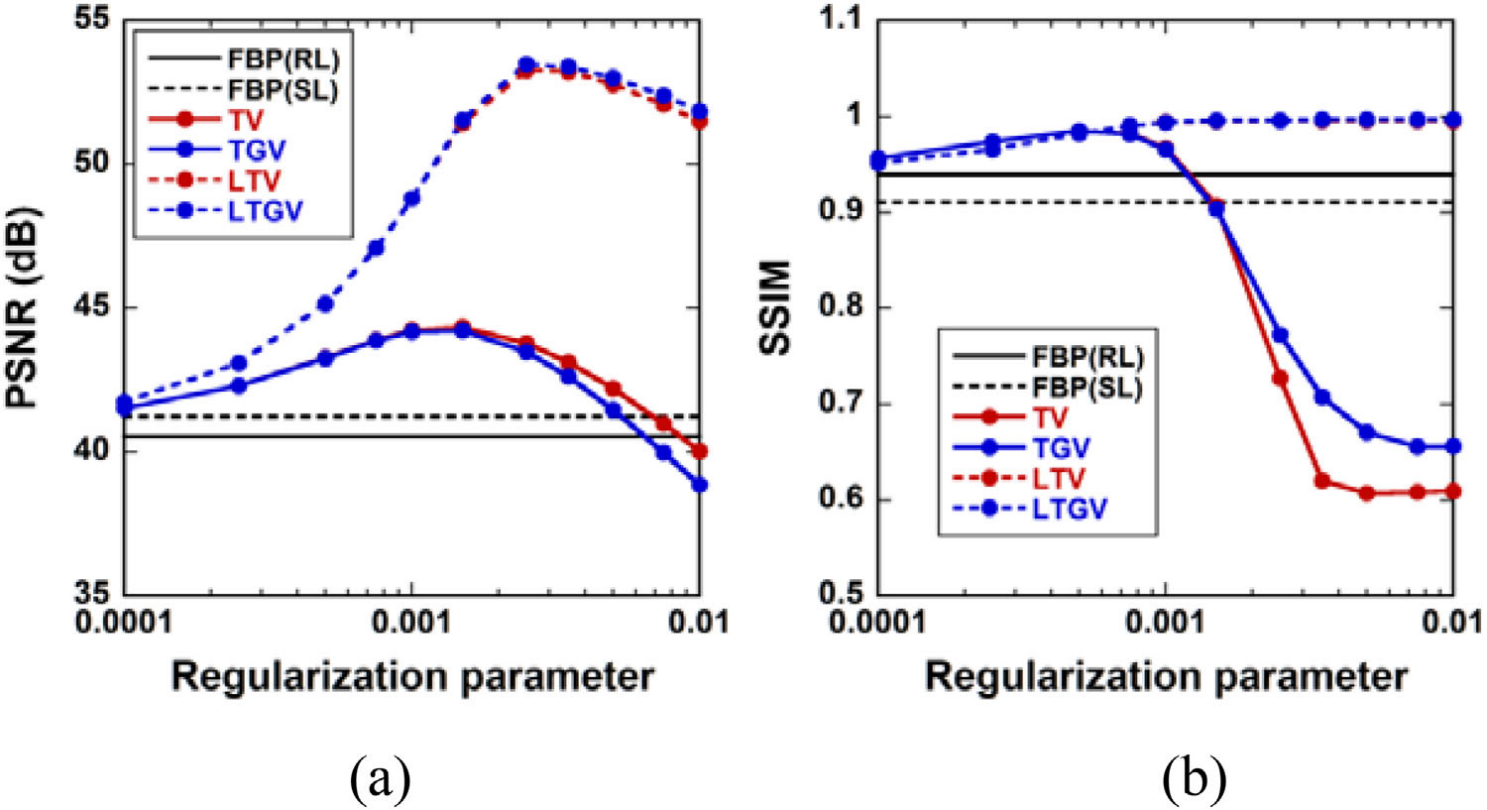
(a) Peak signal‐to‐noise ratio (PSNR) calculated using Equation (9) as a function of the regularization parameter (*α* for TV and LTV, *α*_1_ for TGV and LTGV) value for FBP(RL) (black solid line), FBP(SL) (black dotted line), TV (red solid line), TGV (blue solid line), LTV (red dotted line), and LTGV (blue dotted line). FBP(RL) and FBP(SL) denote the filtered back‐projection method with Ram‐Lak and Shepp‐Logan filters, respectively. TV, TGV, LTV, and LTGV denote the regularization methods described in Sections 2.1.2, 2.1.3, 2.1.4, and 2.1.5, respectively. (b) Structural similarity index measure (SSIM) calculated using Equation (10) as a function of the regularization parameter value. The plots for LTV and LTGV overlap.

Figure 2a shows the PSNR calculated using Equation (9) as a function of the regularization parameter (*α* for TV and LTV, *α*_1_ for TGV and LTGV) value for FBP(RL) (black solid line), FBP(SL) (black dotted line), TV (red solid line), TGV (blue solid line), LTV (red dotted line), and LTGV (blue dotted line). Figure 2b shows the SSIM calculated using Equation (10) as a function of the regularization parameter value. As shown in Figure 2a, the PSNR values for TV and TGV exhibited peaks at *α* and *α*_1_ = 0.001, respectively, whereas those for LTV and LTGV exhibited peaks at *α* and *α*_1_ = 0.0025, respectively. The peaks for LTV and LTGV were higher than those for TV and TGV. Further, the SSIM values for TV and TGV increased gradually until *α* and *α*_1_ were approximately 0.0005, respectively, following which they decreased (Figure 2b). In contrast, those for the LTV and LTGV increased gradually until *α* and *α*_1_ were approximately 0.0015, respectively, following which they plateaued and were close to unity.

**FIGURE 3:**
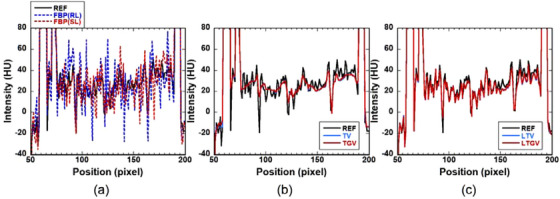
(a) Profiles along the horizontal line passing through the center in the DCE‐CT images at the 20th frame for slice #140 of the digital brain phantom (Figure 1a). Black solid line shows that for the DCE‐CT image reconstructed from noise‐free projection data using FBP(RL) (REF). Blue and red dotted lines show those for the DCE‐CT images reconstructed from noisy projection data using FBP(RL) and FBP(SL), respectively. HU stands for Hounsfield Unit. (b) Profiles for REF (black solid line), TV (blue solid line), and TGV (red solid line). Those for TV and TGV overlap. (c) Profiles for REF (black solid line), LTV (blue solid line), and LTGV (red solid line). Those for LTV and LTGV overlap.

Examples of the horizontal profiles along the line passing through the center in the DCE‐CT images reconstructed using the different methods are shown in Figure 3. These profiles were obtained from DCE‐CT images at the 20th frame for slice #140 of the digital brain phantom (Figure 1a). Figure 3a–c show the profiles for REF (black solid line), FBP(RL) (blue dotted line), and FBP(SL) (red dotted line), those for REF (black solid line), TV (blue solid line), and TGV (red solid line), and those for REF (black solid line), LTV (blue solid line), and LTGV (red solid line), respectively. The values of *α* and *α*_1_ used in the above regularizers were both 0.0025. When using TV and TGV (Figure 3b), the noise was significantly reduced compared to that for FBP(RL) and FBP(SL) (Figure 3a); however, the profiles became blurred and deviated significantly from that for REF. In contrast, when LTV and LTGV were used (Figure 3c), the profiles were nearly identical to that for REF.

**FIGURE 4:**
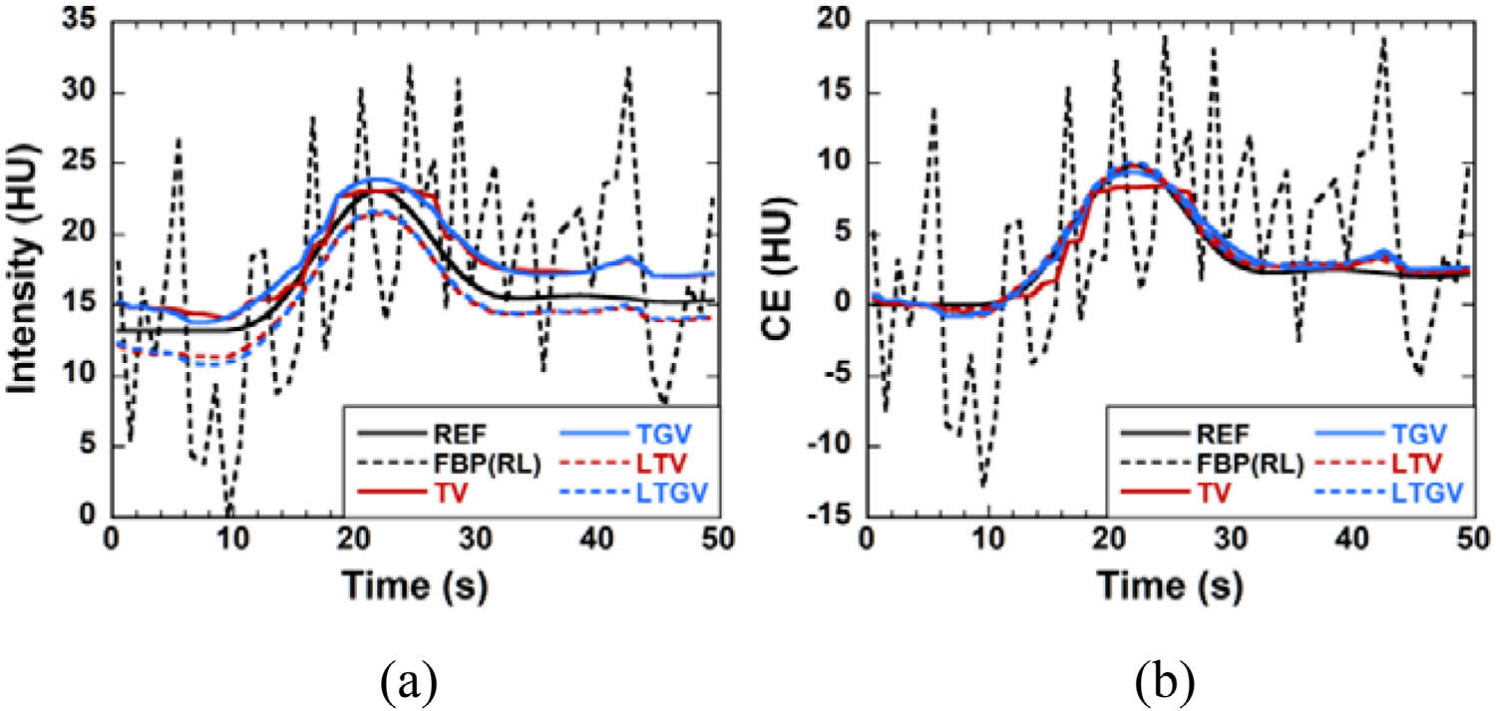
(a) Time‐intensity and (b) contrast‐enhancement (CE) curves at a single pixel in the center of the red circle shown in Figure 1a for REF (black solid line), FBP(RL) (black dotted line). TV (red solid line), TGV (blue solid line), LTV (red dotted line), and LTGV (blue dotted line).

Figure 4a shows examples of time‐intensity curves (TICs) at a single pixel in the center of the red circle illustrated in Figure 1a for REF (black solid line), FBP(RL) (black dotted line), TV (red solid line), TGV (blue solid line), LTV (red dotted line), and LTGV (blue dotted line). Figure 4b shows the CE curves obtained from the TICs shown in Figure 4a. In Figure 4, the plot for FBP(SL) is not shown because it complicates the figure. Although certain noise reduction was obtained with FBP(SL) compared with FBP(RL), it was still considerably noisy. As shown in Figure 4a, the intensities for TV and TGV were greater than that for REF by approximately 1.6 HU, whereas those for LTV and LTGV were smaller by approximately 1.3 HU. In contrast, the CE curves for TGV, LTV, and LTGV nearly overlapped with that for REF (Figure 4b). In addition, staircase artifacts were observed when using TV.

Figure 5a shows the CBF (left column), CBV (middle column), and MTT images (right column) for slice #140 (Figure 1a) obtained from the DCE‐CT images reconstructed using REF (first row), FBP(RL) (second row), and FBP(SL) (third row). Figure 5b displays the CBF images for TV (first row), TGV (second row), LTV (third row), and LTGV (fourth row) with regularization parameter values of 0.001, 0.0025, 0.005, 0.0075, and 0.01 (left to right column), whereas Figure 5c, d show cases for CBV and MTT, respectively. When using TV and LTV, all CBF, CBV, and MTT images significantly improved visually at *α* = 0.0025 compared to those at 0.001 and those for FBP(RL) and FBP(SL), following which they changed with increasing *α*. However, when using TGV and LTGV, they also improved at *α*_1_ = 0.0025 and did not significantly change even at *α*_1_ > 0.0025.

FIGURE 5(a) CBF (left column), CBV (middle column), and MTT images (right column) for slice #140 (Figure 1a) generated from the DCE‐CT images reconstructed using REF (first row), FBP(RL) (second row), and FBP(SL) (third row). (b) CBF, (c) CBV, and (d) MTT images generated from the DCE‐CT images reconstructed using TV (first row), TGV (second row), LTV (third row), and LTGV (fourth row) with regularization parameter values of 0.001, 0.0025, 0.005, 0.0075, and 0.01 (left to right column).

**Figure:**
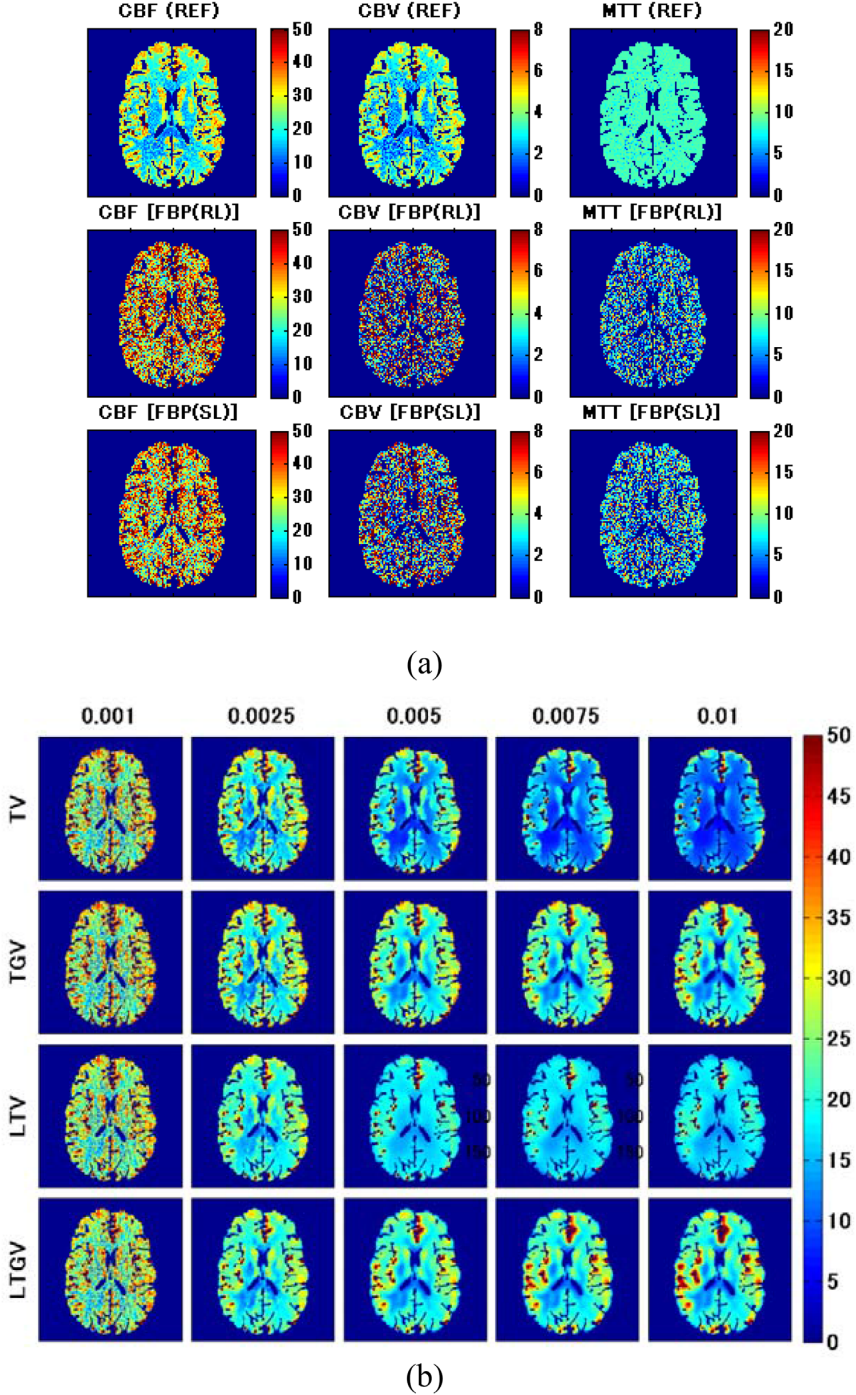

**Figure:**
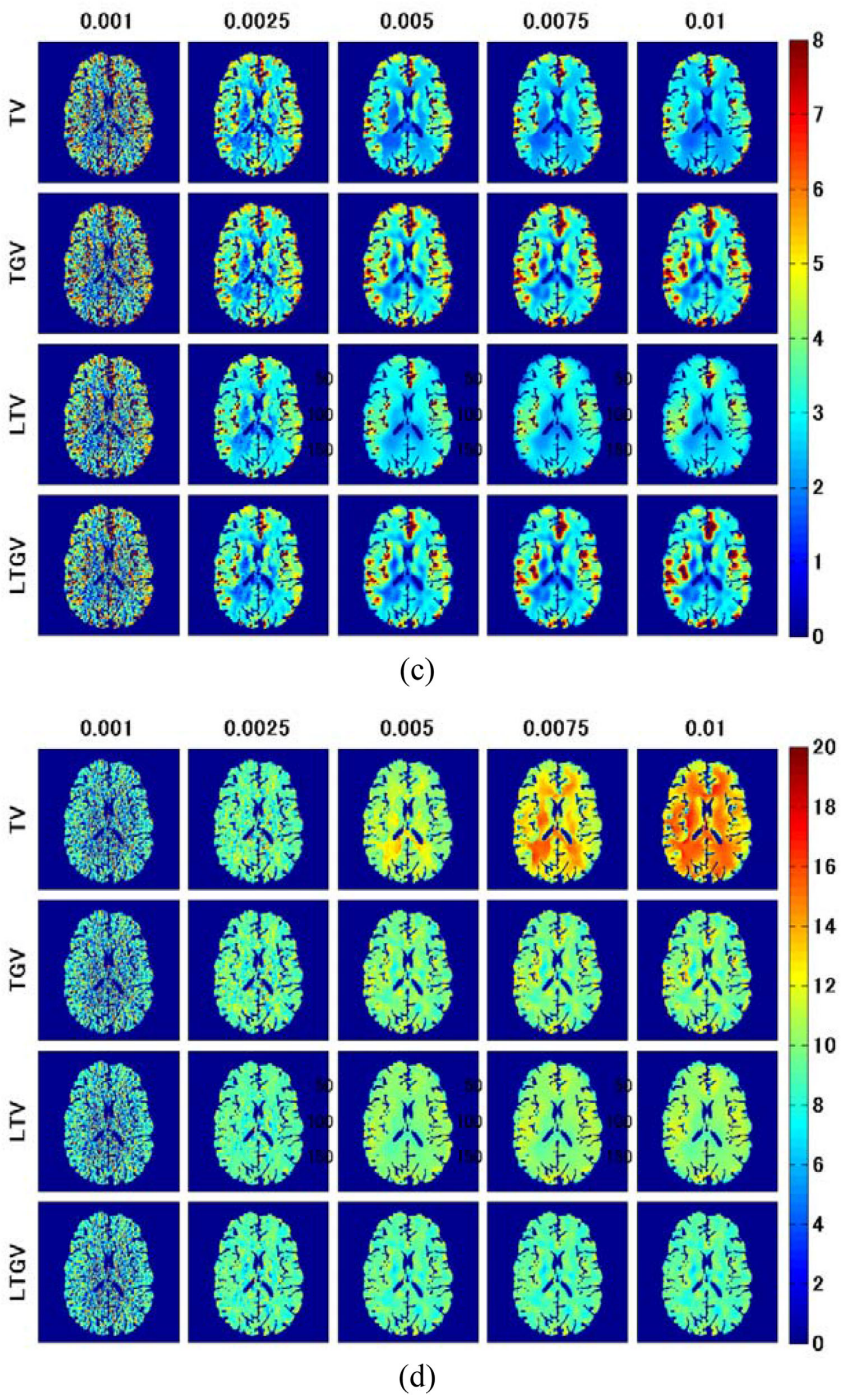

**FIGURE 6:**
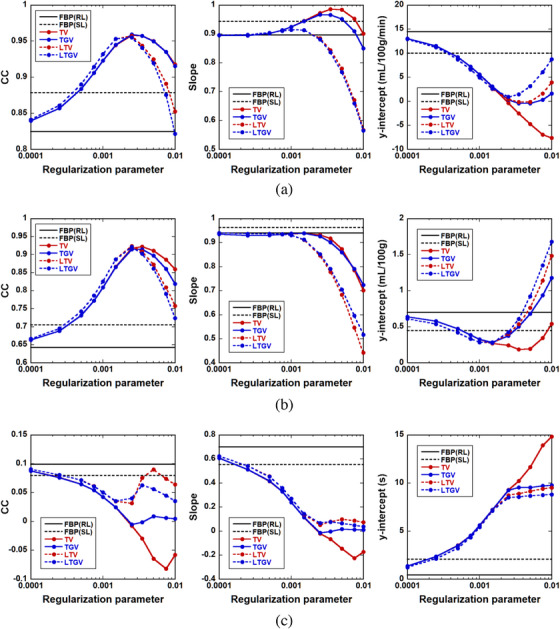
Correlation coefficient (CC) (left panels), slope (middle panels), and y‐intercept (right panels) of the regression equation between the cerebral perfusion parameter values obtained by REF (x‐axis) and those obtained by different methods (y‐axis) as a function of the regularization parameter value. Black solid, black dotted, red solid, blue solid, red dotted, and blue dotted lines show cases for FBP(RL), FBP(SL), TV, TGV, LTV, and LTGV, respectively. (a), (b), and (c) show cases for CBF, CBV, and MTT, respectively.

The results of the linear regression analysis are shown in Figure 6. Figure 6a−c show the CC (left panels), slope (middle panels), and y‐intercept (right panels) as a function of the regularization parameter value for CBF, CBV, and MTT, respectively. As shown in the left panel of Figure 6a, the plots for TV and TGV nearly overlapped and exhibited peaks at 0.0025. In addition, the plots for LTV and LTGV exhibited peaks at 0.0025, following which their CC values decreased with increasing *α* and *α*_1_ more rapidly than those for TV and TGV. Further, the regularization parameter value maximizing the CC approximately coincided with that maximizing the PSNR for the LTV and LTGV (Figure 2a). The slope of CBF for TV was the closest to unity at *α* = 0.003, followed by that for TGV. The slope values for both the LTV and LTGV exhibited peaks at 0.001, which were lower than those for the TV and TGV (middle panel of Figure 6a). In addition, the y‐intercept of CBF for TV monotonically decreased with increasing *α*, whereas those for TGV, LTV, and LTGV were minimized at 0.0035, 0.0035, and 0.0025, respectively, following which they increased again (right panel of Figure 6a). In all regularizers, the CC, slope, and y‐intercept tended to converge to those for the FBP(RL) with decreasing *α* and *α*_1_.

As shown in the left panel of Figure 6b, the CC of the CBV for TV exhibited a peak at *α* = 0.0035, whereas those for the other regularizers exhibited peaks at 0.0025. The slope values of the CBV for TV and TGV and those for LTV and LTGV were approximately constant until 0.0015 and 0.001, respectively, following which they decreased (middle panel of Figure 6b). Further, the y‐intercept of CBV for TV decreased with increasing *α* until 0.0035, following which it increased again, whereas those for the other regularizers were minimized at 0.001−0.0015 (right panel of Figure 6b).

As shown in the left panel of Figure 6c, the CC values of MTT for TV, LTV, and LTGV were the closest to zero at 0.0025, 0.0025, and 0.0015, respectively, whereas that for TGV was almost constant after it became closest to zero at 0.0025. In addition, the slope of MTT for TV decreased with increasing *α* until 0.0075, whereas those for the other regularizers were minimized at 0.0025, following which they were approximately constant (middle panel of Figure 6c). When using TGV, both the CC and slope were approximately zero at *α*_1_ > 0.0025, thereby reflecting the homogeneous MTT distribution. The y‐intercept of MTT for TV monotonically increased with increasing *α*, whereas those for the other regularizers also increased until 0.0025. Thereafter, they plateaued (right panel of Figure 6c). This plateau value roughly matched the mean MTT value in the MTT image for REF (8.54 s) (Figure 5a).

CBF, CBV, and MTT images of an ischemic lesion (Figure 1b) are shown in Figure 7. Similar to Figure 5, Figure 7a shows the CBF (left column), CBV (middle column), and MTT images (right column) for REF (first row), FBP(RL) (second row), and FBP(SL) (third row). Figure 7b–d show the CBF, CBV, and MTT images, respectively, for TV (first row), TGV (second row), LTV (third row), and LTGV (fourth row) with regularization parameter values of 0.001, 0.0025, 0.005, 0.0075, and 0.01 (left to right column). The dependencies of the CBF, CBV, and MTT images on the regularization parameter value were similar to those shown in Figure 5. When using TGV and LTGV, a prolonged MTT in the penumbra was more clearly demonstrated than when using TV and LTV at *α* and *α*_1_ *>* 0.0025.

FIGURE 7(a) CBF (left column), CBV (middle column), and MTT images (right column) for slice #152 (Figure 1b) obtained by REF (first row), FBP(RL) (second row), and FBP(SL) (third row). (b) CBF, (c) CBV, and (d) MTT images obtained by TV (first row), TGV (second row), LTV (third row), and LTGV (fourth row) with regularization parameter values of 0.001, 0.0025, 0.005, 0.0075, and 0.01 (left to right column).

**Figure:**
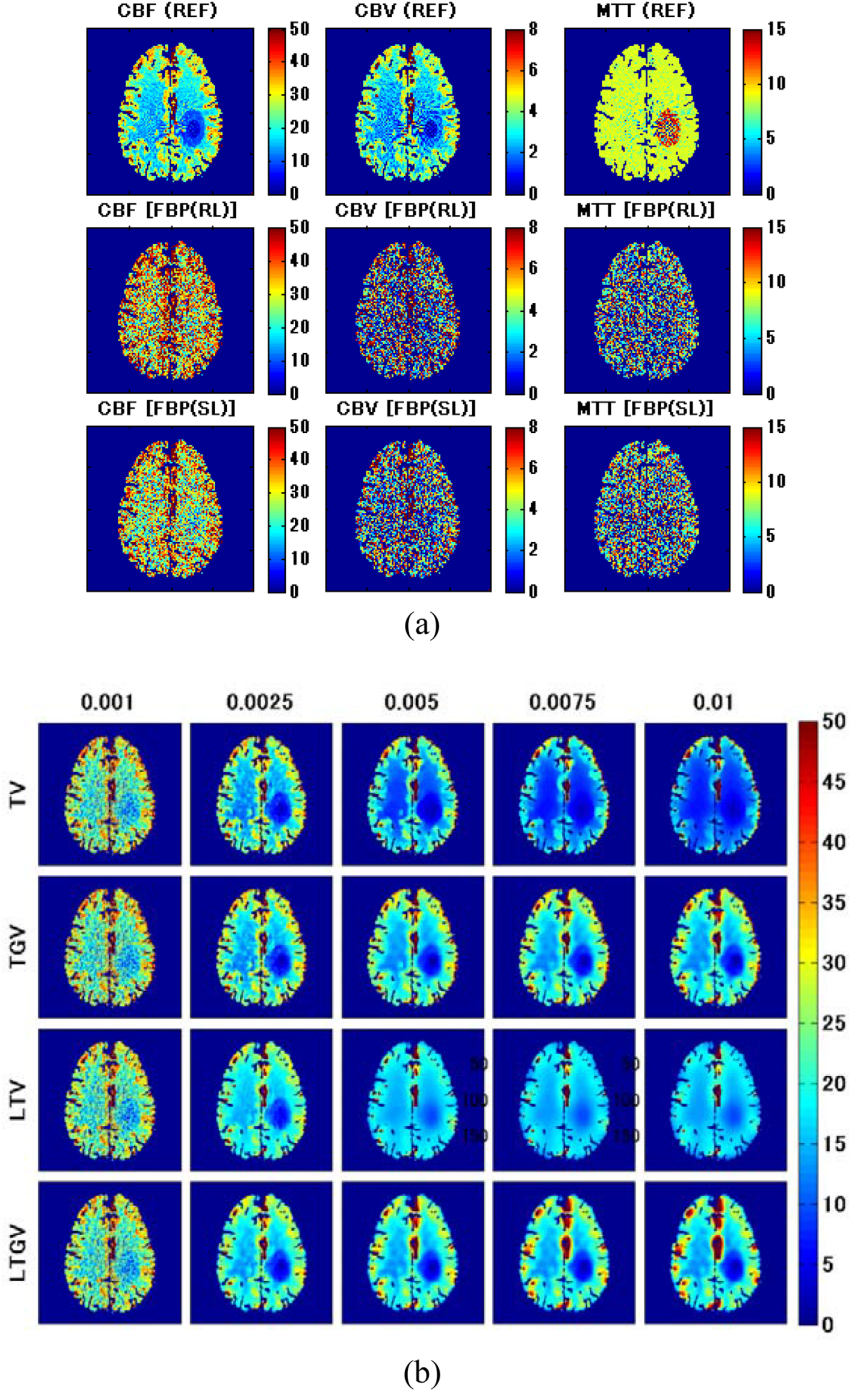

**Figure:**
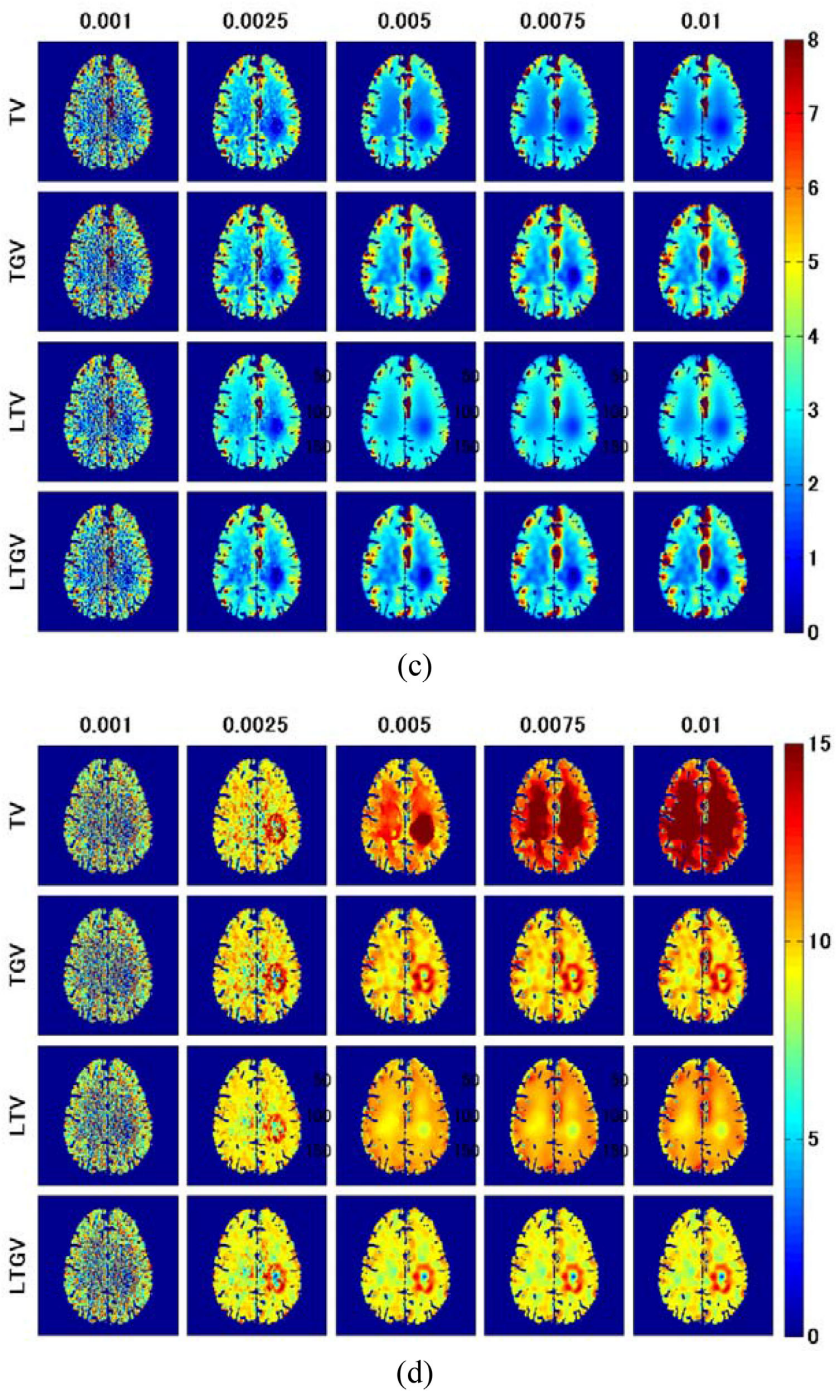

**FIGURE 8:**
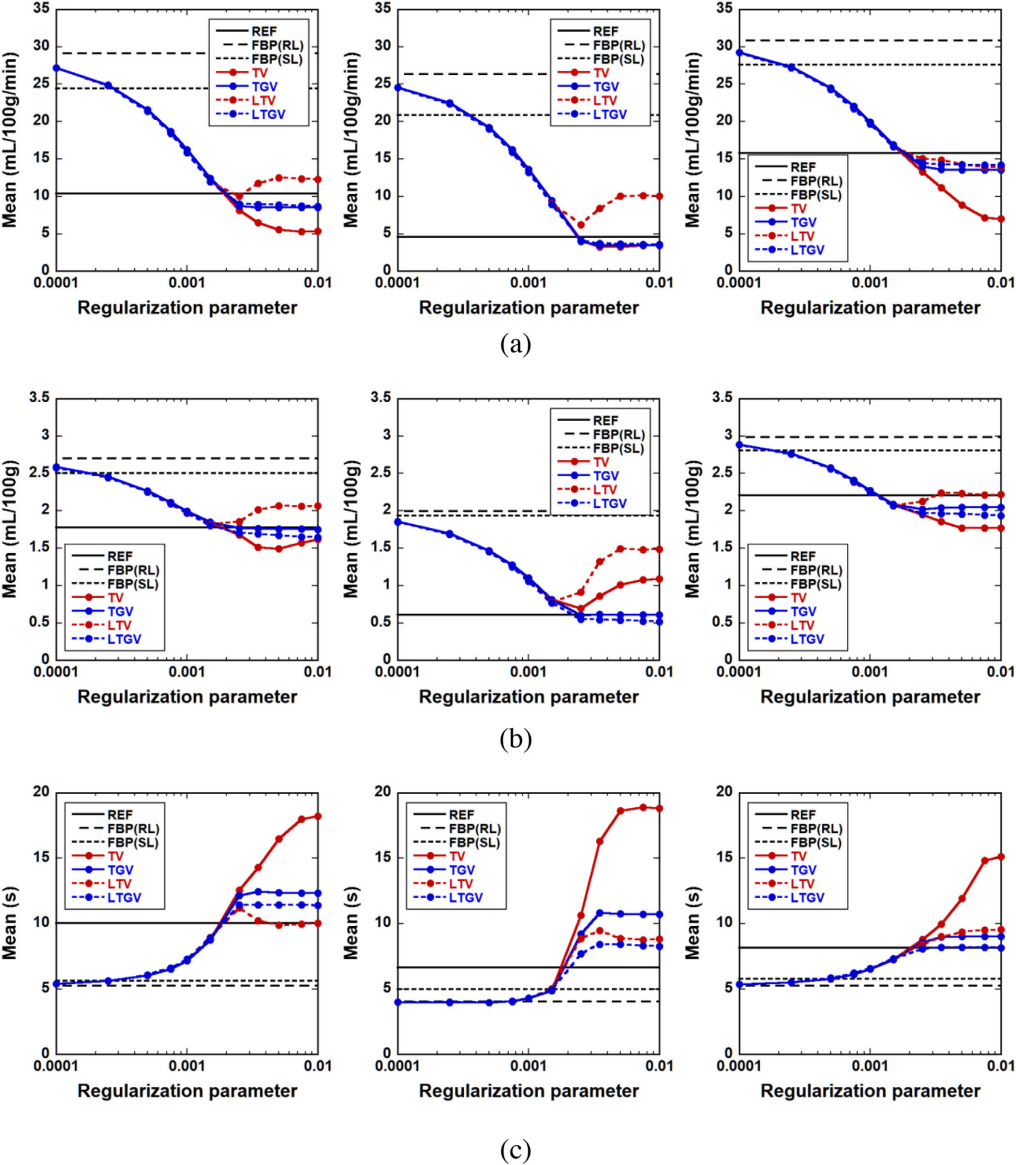
Mean of (a) CBF, (b) CBV, and (c) MTT in the penumbra (left column), ischemic core (middle column), and contralateral normal region (right column) as a function of the regularization parameter value for REF (black solid line), FBP(RL) (black dashed line), FBP(SL) (black dotted line), TV (red solid line), TGV (blue solid line), LTV (red dotted line), and LTGV (blue dotted line).

**FIGURE 9:**
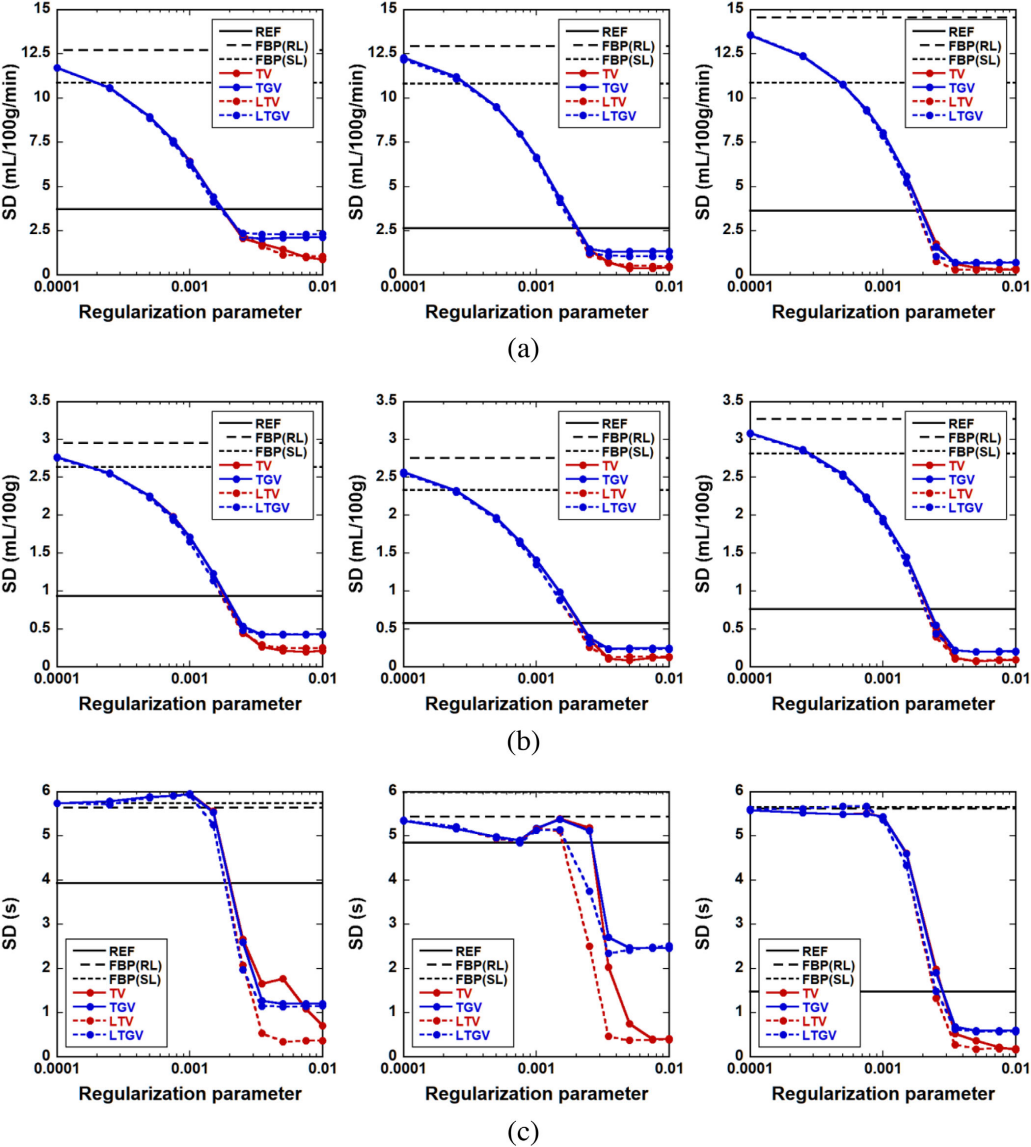
Standard deviation (SD) of (a) CBF, (b) CBV, and (c) MTT in the penumbra (left column), ischemic core (middle column), and contralateral normal region (right column) as a function of the regularization parameter value for REF (black solid line), FBP(RL) (black dashed line), FBP(SL) (black dotted line), TV (red solid line), TGV (blue solid line), LTV (red dotted line), and LTGV (blue dotted line).

The results of ROI analysis are shown in Figures 8 and 9. Figure 8a shows the mean CBF values in the penumbra (left column), ischemic core (middle column), and contralateral normal region (right column) as a function of the regularization parameter value for REF (black solid line), FBP(RL) (black dashed line), FBP(SL) (black dotted line), TV (red solid line), TGV (blue solid line), LTV (red dotted line), and LTGV (blue dotted line). As shown in the left panel of Figure 8a, the mean CBF value in the penumbra for TV monotonically decreased with increasing *α*, whereas those for the other regularizers decreased until 0.0025, following which those for TGV and LTGV plateaued and that for LTV increased again and then plateaued. The mean CBF values in the ischemic core for TV, TGV, and LTGV decreased until 0.0025, following which they plateaued. In contrast, that for LTV also decreased until 0.0025, following which it increased again and then plateaued (middle panel of Figure 8a). Furthermore, the mean CBF value in the contralateral normal region for TV monotonically decreased, whereas those for the other regularizers decreased until 0.0025, following which they plateaued (right panel of Figure 8a).

Figure 8b shows the cases for CBV. As shown in the left panel of Figure 8b, the mean CBV value in the penumbra for TV decreased with increasing *α* until 0.005, following which it increased again. In contrast, those for TGV and LTGV decreased until 0.0025, following which they tended to plateau. Further, the value of LTV was minimized at 0.0015, following which it increased again and then plateaued. The mean CBV values in the ischemic core for TV and LTV were minimized at 0.0025 and 0.0015, respectively, following which they increased again, whereas those for TGV and LTGV were minimized at 0.0025, following which they plateaued (middle panel of Figure 8b). Moreover, the dependency of the men CBV value on the regularization parameter value in the contralateral normal region was similar to that in the penumbra (right panel of Figure 8b).

The results of the MTT assay are shown in Figure 8c. As shown in the left panel of Figure 8c, the mean MTT value in the penumbra for TV monotonically increased with increasing *α*, whereas those for TGV and LTGV increased until 0.0025, following which they plateaued. In addition, the value of LTV increased until 0.0025, following which it decreased and then plateaued. Similar behaviors were observed in the ischemic core and contralateral normal region (middle and right panels of Figure 8c).

As in Figure 8, the SD values in the penumbra, ischemic core, and contralateral normal region for different methods are displayed in Figure 9. Figure 9a–c show the cases of CBF, CBV, and MTT, respectively. As shown in the left panel of Figure 9a, the SD values of CBF in the penumbra for TV and LTV monotonically decreased with increasing *α*. Except for this case, the SD values of CBF and CBV for all regularizers monotonically decreased until 0.0025, following which they tended to plateau (Figure 9a, b). As shown in the left panel of Figure 9c, the SD values of MTT in the penumbra for all regularizers were approximately constant until 0.0015, following which they decreased until 0.0035 and then plateaued. Similar behaviors were observed in the ischemic core and contralateral normal region (middle and right panels of Figure 9c). Moreover, the SD values at the plateau for LTV were the lowest and closest to zero in all the regions (Figure 9c).

The results of the clinical study are shown in Figure 10. The first row of Figure 10a shows the CBF (left column), CBV (middle column), and MTT images (right column) obtained by REF; that is, the images obtained from the normal‐dose DCE‐CT images reconstructed using FBP(RL). Further, the second and third rows of Figure 10a show those obtained from the low‐dose DCE‐CT images reconstructed using FBP(RL) and FBP(SL), respectively. Figure 10b shows the CBF images for TV (first row), TGV (second row), LTV (third row), and LTGV (fourth row) with regularization parameter values of 0.001, 0.0025, 0.005, 0.0075, and 0.01 (left to right column). As in Figure 10b, the CBV and MTT images are shown in Figure 10c, d, respectively. When using TV, the image quality improved at *α* = 0.0025, following which the spatial resolution significantly deteriorated with increasing *α*. Moreover, the quality of the images obtained by TGV also improved at *α*_1_ *=* 0.0025, and the spatial resolution deteriorated with increasing *α*_1_; however, it was lesser than in the case of TV. In addition, the qualities of the images obtained by LTV and LTGV also improved at 0.0025, following which they were preserved. The spatial resolution of LTGV was better than that of LTV.

FIGURE 10(a) CBF (left column), CBV (middle column), and MTT images (right column) obtained in a patient with occlusive cerebrovascular disease. The first row shows those generated from the normal‐dose DCE‐CT images reconstructed using FBP(RL) (REF). The second and third rows show those generated from the low‐dose DCE‐CT images reconstructed using FBP(RL) and FBP(SL), respectively. (b) CBF, (c) CBV, and (d) MTT images generated from the low‐dose DCE‐CT images reconstructed using TV (first row), TGV (second row), LTV (third row), and LTGV (fourth row) with regularization parameter values of 0.001, 0.0025, 0.005, 0.0075, and 0.01 (left to right column).

**Figure:**
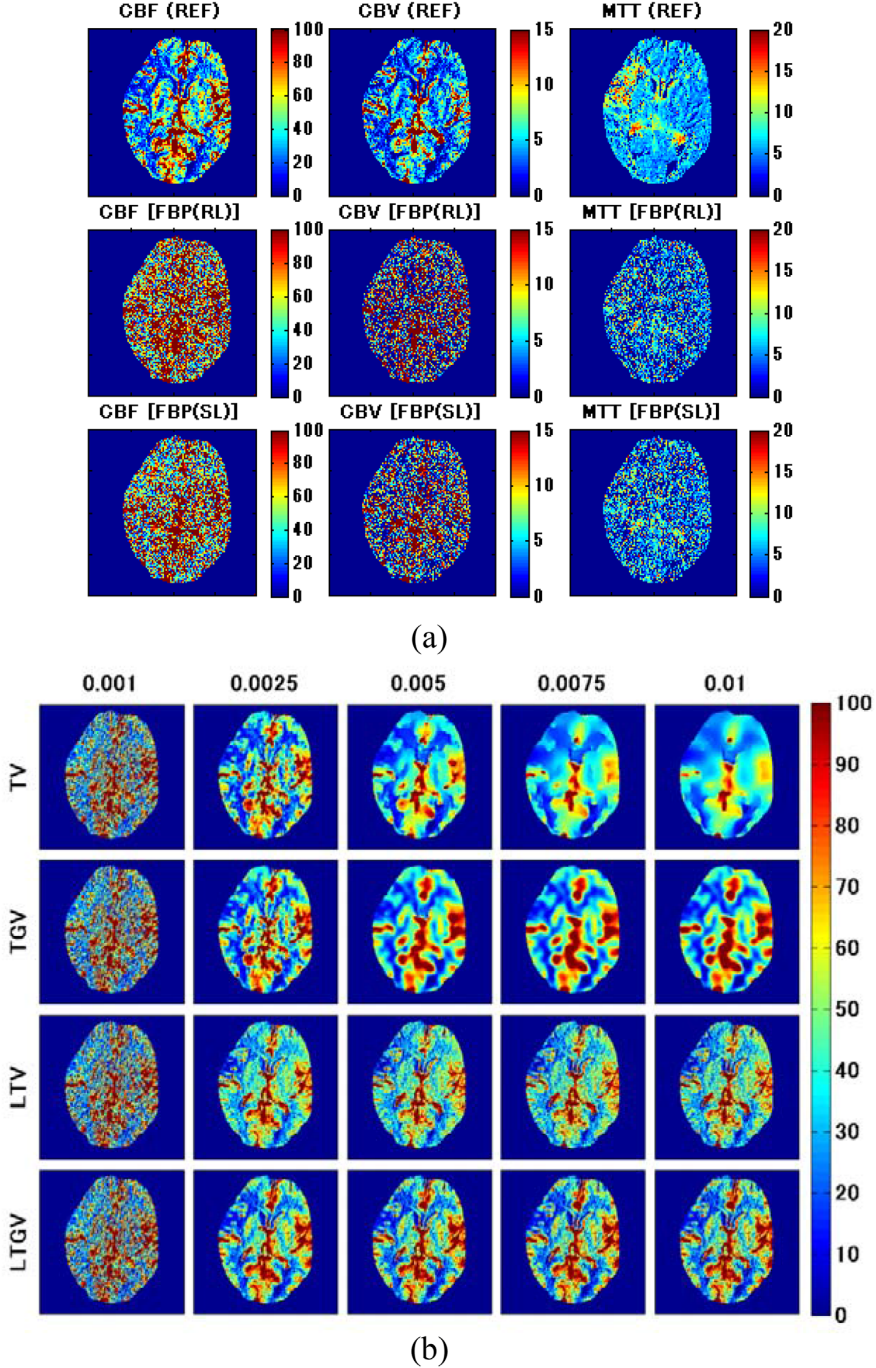

**Figure:**
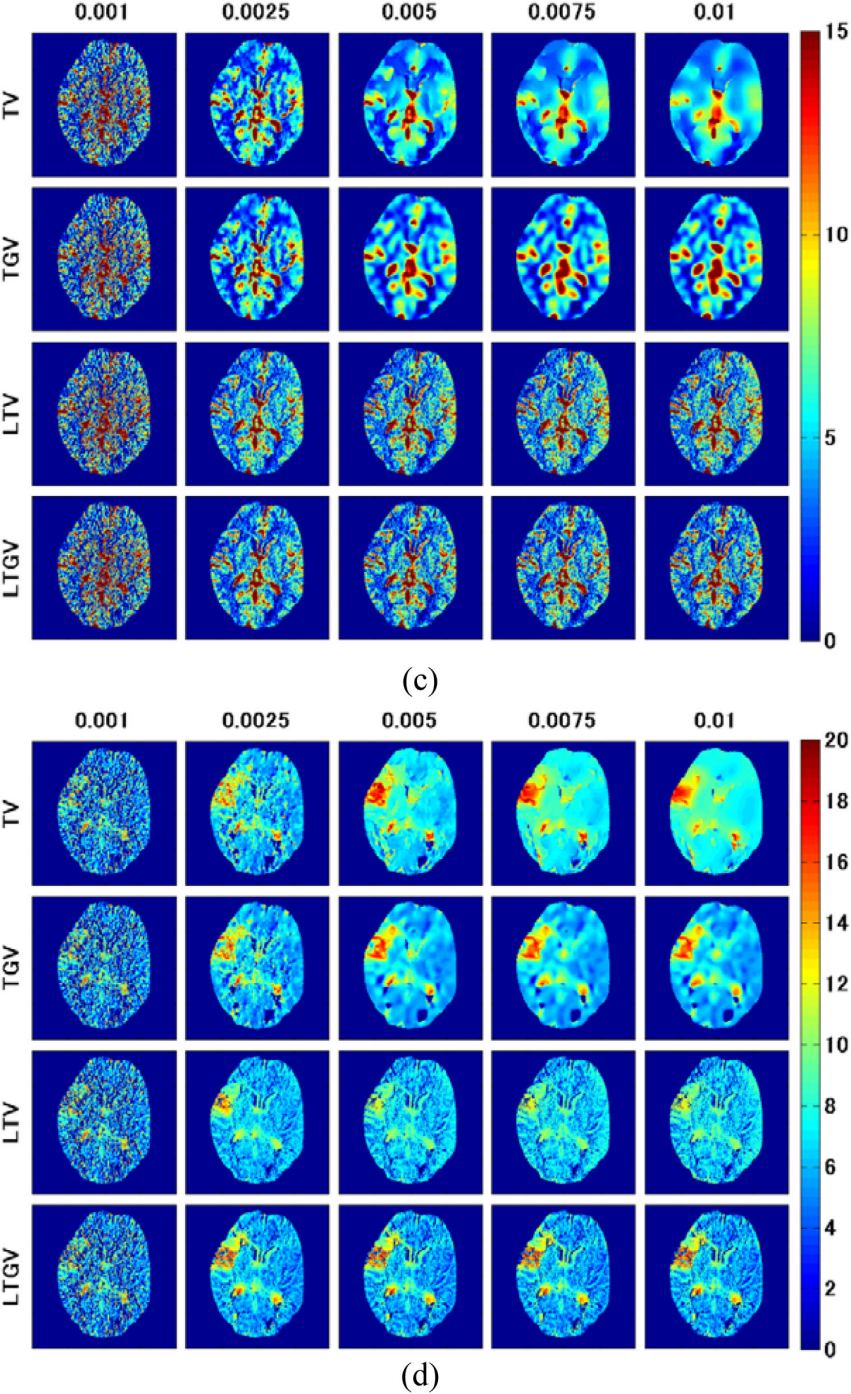

## 4 DISCUSSION

This study quantitatively investigated the performance of different simultaneous spatial and temporal regularization methods when applied to low‐dose DCE‐CT cerebral perfusion studies by analyzing the quality of the reconstructed DCE‐CT and cerebral perfusion parameter (CBF, CBV, and MTT) images generated from them. The results indicate that this study provides a better understanding of the characteristics of regularizers in terms of the resulting image quality and accuracy of perfusion parameter estimation. Moreover, it is expected to aid in the selection of a suitable regularizer for low‐dose DCE‐CT cerebral perfusion studies.

The TGV expressed by Equation (3) can be considered a generalization of TV.^21^ As shown in Figure 4, when using the TV, staircase artifacts were observed, whereas such artifacts disappeared when using the TGV. This is because only the first derivative of the image was considered in TV (Equation 1), whereas the higher‐order derivative of the image was used in TGV (Equation 3). Thus, TGV appears more suitable for modeling image‐intensity variations in smooth regions than TV and is more effective in preserving the edge information of the image without causing staircase artifacts.^23^

When using LTV and LTGV, DCE‐CT images are decomposed into L and S components using LRSD. In the LTV, the NN of the L component and the TV of the S component are minimized (Equation 4), whereas the TGV of the S component in addition to the NN of the L component are minimized in the LTGV (Equation 5). As described by Otazo et al.,^26^ the L component of dynamic images can model the temporally correlated (time‐coherent) background (static features), including anatomical information, and the S component can model the dynamic features. Therefore, the NN minimization of the L component is expected to aid in the preservation of static features owing to the reduction of background artifacts. Whereas it is expected that the TV minimization of the S component would facilitate noise reduction while the TGV minimization would result in both noise reduction and edge preservation. Thus, it appears that these effects in LTV and LTGV produced higher PSNR and SSIM than those for TV and TGV (Figure 2). Although staircase artifacts were observed when using TV as described above, they disappeared when the TV was combined with LRSD (LTV) (Figure 4), suggesting that LRSD was also effective for reducing these artifacts in dynamic images.

As shown in Murase et al.,^35^ when the NN minimization was applied to dynamic images without separating them into L and S components (referred to as the NN method in Murase et al.^35^ or simply low‐rank decomposition (LRD) in general), the dynamic images were significantly distorted mainly in the image‐intensity direction, resulting in accuracy deterioration of kinetic parameter estimation.^35^ Thus, we used LRSD rather than LRD in this study.

In the present simulation results, TGV generally showed the best performance in terms of the accuracy of cerebral perfusion parameter estimation, followed by LTGV (Figures 5, 6, 7, 8, 9). This indicates that these methods are useful for decreasing radiation exposure during DCE‐CT perfusion studies without significantly reducing the accuracy of perfusion parameter estimation. In contrast, the performance of TV was significantly different from those of the other regularizers, whereas that of LTV was similar to those of TGV and LTGV. This implies that the LRSD used in LTV improved the performance of TV.

Although both PSNR and SSIM for LTV and LTGV were higher than those for TV and TGV (Figure 2), the corresponding CC and slope values of CBF and CBV were lower than those for TV and TGV in case of large *α* and *α*_1_ (Figure 6a, b). As previously described, when generating cerebral perfusion parameter images using Equations (6)–(8), the concentration of CA was replaced by CE, which was calculated by subtracting the pre‐contrast (baseline) image from DCE‐CT images. Thus, the above finding may be becuase of the fact that although the static feature of DCE‐CT images is presevred when using LTV and LTGV, the image distortion owing to NN minimization of the L component of DCE‐CT images increased, particularly in regions with extremely high CBF and CBV, such as blood vessels. Consequently, its effect propagated to the perfusion parameter estimation through the baseline subtraction when the regularization paremeter value is large.

Visual inspection of the generated perfusion parameter images (Figures 5 and 7) indicated that the noise reduction by TGV and LTGV was lower than that by TV and LTV when *α* and *α*_1_ were greater than approximately 0.0025. This was also confirmed by the fact that the SD values for TGV and LTGV were greater than those for TV and LTV (Figure 9). However, the stability of perfusion parameter estimation for TGV and LTGV was superior to that for TV and LTV, because the mean CBF, CBV, and MTT values obtained by them were approximately constant. In addition, their variations were much smaller than those for TV and LTV when *α* and *α*_1_ were greater than approximately 0.0025 (Figure 8). This was also confirmed by visual inspection of the images (Figures 5 and 7). These results suggest that TGV and LTGV are more insensitive and flexible for the selection of regularization parameter values than TV and LTV, and the ranges of their available regularization parameter values are larger than those of TV and LTV. Thus, TGV and LTGV appear to be useful for reducing the time and effort required for tuning regularization parameter values. This feature is particularly important in practical applications, because in contrast to the simulation studies, the reference used for evaluating the image quality and accuracy of perfusion parameter estimation is unknown.

Kikuchi et al.^46^ demonstrated that MTT images may be used to evaluate the extent of cerebral perfusion reserve impairment in patients with occlusive cerebrovascular disease because the increased MTT correlated well with decreased cerebral perfusion reserve determined when performing Xe‐133 single photon emission computed tomography examinations before and after administration of acetazolamide. A prolonged MTT in the ischemic region was also observed in our clinical data (Figure 10). Thus, MTT images can aid in determining treatment strategies and predicting the outcomes of patients with cerebrovascular diseases. As shown in our simulation results, when using TGV and LTGV, the MTT images were homogeneous in the brain without any lesions (Figure 5d), whereas in the case with an ischemic lesion, the prolonged MTT in the penumbra was more clearly demonstrated compared to those obtained by the other methods, even when the regularization parameter value was large (Figure 7d). These results suggest that these methods are useful for evaluating cerebral perfusion reserve impairments.

In the clinical study, the quality of the cerebral perfusion parameter images was the best when *α* and *α*_1_ were 0.0025 for all regularizers (Figure 10). When *α* was greater than 0.0025, the spatial resolution of the images obtained by TV significantly deteriorated with increasing *α*. In contrast, it did not change significantly when LTGV was used. These findings were consistent with those obtained in the simulation study (Figures 5 and 7). However, certain inconsistencies between the clinical and simulation results were observed; that is, the spatial resolution of the images obtained by TGV with *α*_1_ geater than 0.0025 decreased with increasing *α*_1_, although not to the extent of that in the case for TV (Figure 10). As previously described, we avoided scanning a patient twice from an ethical perspective and generated low‐dose DCE‐CT images from normal‐dose DCE‐CT images in the same manner as in the simulation study. Thus, the variation in the background, noise level, and noise distribution may differ in both studies, which may be the main reason for the above inconsistency.

In this study, primarily, cases with *I*_0_ of 2.5 × 10^5^ were investigated. When *I*_0_ was varied, the regularization parameter value maximizing PSNR (Figure 2a) decreased with increasing *I*_0_ and vice versa. When using TV and TGV, it was approximately 0.0025, 0.001, and 0.00075 for *I*_0_ of 1.0 × 10^5^, 2.5 × 10^5^, and 5.0 × 10^5^, respectively. Further, when using LTV and LTGV, it was approximately 0.005, 0.0025, and 0.0015 for *I*_0_ of 1.0 × 10^5^, 2.5 × 10^5^, and 5.0 × 10^5^, respectively. However, the performance of the above methods was similar to that obtained in this study within the studied range of *I*_0_ (data not shown). Thus, the findings of this study can also be applied to these cases.

We also assumed that the regularization parameter (*α* and *α*_1_) values were constant regardless of space and time. However, their optimal values may vary depending on local image intensity and noise level. Recently, Shen et al.^47^ attempted to use deep reinforcement learning for intelligent parameter tuning in iterative CT image reconstruction. When combining the present regularization methods with their intelligent parameter tuning method,^47^ pixel‐wise and intellectual adjustment of regularizers may be possible, and this investigation will be handled in future studies. A comparative study with other AI‐based methods is planned.

## 5 CONCLUSIONS

We quantitatively investigated the performance of different simultaneous spatial and temporal regularization methods when applied to low‐dose DCE‐CT cerebral perfusion studies. Our results suggest that TGV and LTGV can improve the accuracy of cerebral perfusion parameter estimation using low‐dose DCE‐CT. This study can be used to improve the understanding of the performance of simultaneous spatial and temporal regularizers, and is expected to aid in the selection of a suitable regularizer for cerebral perfusion studies using low‐dose DCE‐CT.

## AUTHOR CONTRIBUTIONS

Kenya Murase contributed to the conceptualization, methodology, data curation, software, original draft preparation, and writing. Atsushi Nakamoto and Noriyuki Tomiyama contributed to the revising and editing.

## CONFLICT OF INTEREST STATEMENT

The authors declare no competing interest.

## 1

The primal‐dual (PD) method is a preconditioned version of the alternating direction method of multipliers, and the details are described elsewhere.^32^, ^33^ The PD method has been widely used to solve the following convex‐concave saddle‐point problem:

(A1)
$$\min_{x\in X}\;\max_{y\in Y}\left(\left\langle Kx,y\right\rangle +G\left(x\right)-F^{*}\left(y\right)\right),$$

where ***X*** and ***Y*** are Hilbert spaces, ***K*** is a linear operator, *F* and *G* are convex functions, and ⟨·,·⟩ denotes the inner product. Further, $F^{*}\left(y\right)$ is the conjugate of *F*(***y***), which can be obtained using the Legendre transform as follows^32^:

(A2)
$$F^{*}\left(y\right)=\max_{y^{\prime }}\left\{\left\langle y,y^{\prime }\right\rangle -F\left(y^{\prime }\right)\right\}\;.$$

In this study, we adopted the Chambolle–Pock (CP) algorithm^48^ to solve Equation (A1). The CP algorithm is a first‐order PD hybrid‐gradient method for non‐smooth convex optimization problems with a known saddle‐point structure and involves iterative alternation of a gradient‐like ascent in the dual variable ***y*** and a gradient‐like descent in the primal variable ***x***. In addition, an over‐relaxation in ***x*** is performed as follows^32^:

(A3)
$$\begin{array}{c} y_{k+1}=\mathrm{pro}x_{\sigma }\left[F^{*}\right]\left(y_{k}+\sigma K{\bar{x}}_{k}\right),\\ x_{k+1}=\mathrm{pro}x_{\tau }\left[G\right]\left(x_{k}-\tau K^{*}y_{k+1}\right),\\ {\bar{x}}_{k+1}=2x_{k+1}-x_{k}, \end{array}$$

where *k* is the iteration number, σ and τ denote the step sizes, and $prox_{\sigma }$ and $prox_{\tau }$ are the proximal operators defined by

(A4)
$$\begin{array}{c} \mathrm{pro}x_{\sigma }\left[F^{*}\right]\;\left(z\right)=\underset{z^{\prime }}{\mathrm{argmin}}\left\{F^{*}\left(z^{\prime }\right)+\frac{∥z-z^{\prime }{∥}_{F}^{2}}{2\sigma }\right\}. \end{array}$$

When applying the CP algorithm to the TGV, ***x*** and ***y*** are expressed as [***x*** ***v***]^*T*^ and [***p q r***]^*T*^, respectively, and ***K*** is expressed as

(A5)
$$K\;=\left(\begin{array}{cc} A & \;0\\ \nabla_{3} & \;-I\\ 0 & \;E \end{array}\right)\;,$$

where ***I*** denotes an identity matrix. *F* and *G* are expressed as

(A6)
$$\begin{array}{c} F\left(p,q,r\right)=\frac{1}{2}{\left\|p-b\right\|}_{F}^{2}+\alpha_{1}{\left\|q\right\|}_{1}+\alpha_{0}{\left\|r\right\|}_{1},\\ G\left(x,v\right)=0. \end{array}$$

When using Equation (A2), $F^{*}\left(p,q,r\right)$ is expressed as

(A7)
$$\begin{array}{c} F^{*}\left(p,q,r\right)=\left\langle p,b\right\rangle \;+\frac{1}{2}∥p{∥}_{F}^{2}+\delta_{\mathrm{Box}\left(\alpha_{1}\right)}\left(q\right)+\delta_{\mathrm{Box}\left(\alpha_{0}\right)}\left(r\right), \end{array}$$

where $\delta_{Box(\alpha_{1})}$ and $\delta_{Box(\alpha_{0})}$ are indicator functions defined by^32^

(A8)
$$\delta_{\mathrm{Box}\left(\alpha \right)}\left(z\right)=\left\{\begin{array}{c} 0\;∥z{∥}_{\infty }\leq \alpha \\ \infty \;∥z{∥}_{\infty }>\alpha \end{array}\right.\;.$$

When using Equations (A6) and (A7), the iterative procedure expressed by Equation (A3) can be reduced to^27^

(A9)
$$\begin{array}{ccc} \;p_{k+1} & = & \frac{p_{k}+\sigma \left(A{\bar{x}}_{k}-b\right)}{1+\sigma },\\ q_{k+1} & = & P_{\alpha_{1}}\left(q_{k}+\sigma \left(\nabla_{3}{\bar{x}}_{k}-{\bar{v}}_{k}\right)\right),\\ r_{k+1} & = & P_{\alpha_{0}}\left(r_{k}+\sigma E\left({\bar{v}}_{k}\right)\right),\\ x_{k+1} & = & x_{k}-\tau \left(A^{*}p_{k+1}-\mathrm{di}v_{1}\left(q_{k+1}\right)\right),\\ v_{k+1} & = & v_{k}+\tau \left(q_{k+1}+\mathrm{di}v_{2}\left(r_{k+1}\right)\right),\\ {\bar{x}}_{k+1} & = & 2x_{k+1}-x_{k},\\ {\bar{v}}_{k+1} & = & 2v_{k+1}-v_{k}, \end{array}$$

where $\mathrm{di}v_{1}=-\nabla_{3}^{*}$, $\mathrm{di}v_{2}=-E^{*}$, and $P_{\alpha }$ is the projection operator defined by^27^, ^32^

(A10)
$$P_{\alpha }\left(z\right)=\frac{z}{\max\left(1,\;\left|z\right|/\alpha \right)}.$$

In the case of TV, α_1_ is replaced by α, ***x***, ***y***, and ***K*** are expressed as ***x***, [***p q***]^*T*^, and [***A*** ∇_3_]^*T*^, respectively, and the equations including ***r*** and ***v*** in Equation (A9) are neglected.

When applying the CP algorithm to LTGV, ***x*** and ***y*** are expressed as [***L S v***]^*T*^ and [***p q r***]^*T*^, respectively, and ***K*** is expressed as

(A11)
$$K\;=\left(\begin{array}{ccc} A & \;A & \;0\\ 0 & \;\nabla_{3} & \;-I\\ 0 & \;0 & \;E \end{array}\right)\;.$$

*F* and *G* are expressed as

(A12)
$$\begin{array}{c} F\left(p,q,r\right)=\frac{1}{2}{\left\|p-b\right\|}_{F}^{2}+\alpha_{1}{\left\|q\right\|}_{1}+\alpha_{0}{\left\|r\right\|}_{1},\\ G\left(L,S,v\right)=\beta {\left\|L\right\|}_{*}\cdot \end{array}$$

As in Equation (A9), when using Equations (A7) and (A12), the primal and dual variables are iteratively updated as follows:

(A13)
$$\begin{array}{ccc} p_{k+1} & = & \frac{p_{k}+\sigma \left(A\left({\bar{L}}_{k}+{\bar{S}}_{k}\right)-b\right)}{1+\sigma },\\ q_{k+1} & = & P_{\alpha_{1}}\left(q_{k}+\sigma \left(\nabla_{3}{\bar{S}}_{k}-{\bar{v}}_{k}\right)\right),\\ r_{k+1} & = & P_{\alpha_{0}}\left(r_{k}+\sigma E\left({\bar{v}}_{k}\right)\right),\\ L_{k+1} & = & S_{\tau \beta }\left(L_{k}-\tau A^{*}p_{k+1}\right),\\ S_{k+1} & = & S_{k}-\tau \left(A^{*}p_{k+1}-\mathrm{di}v_{1}\left(q_{k+1}\right)\right),\\ v_{k+1} & = & v_{k}+\tau \left(q_{k+1}+\mathrm{di}v_{2}\left(r_{k+1}\right)\right),\\ {\bar{L}}_{k+1} & = & 2L_{k+1}-L_{k},\\ {\bar{S}}_{k+1} & = & 2S_{k+1}-S_{k},\\ {\bar{v}}_{k+1} & = & 2v_{k+1}-v_{k}. \end{array}$$

$S_{\tau \beta }$ in Equation (A13) is the singular value shrinkage operator defined by^49^

(A14)
$$S_{\beta }\left(z\right)=\;US_{\beta }\left(\Sigma \right)V^{T},$$

where ***U***, ***Σ***, and ***V*** denote the matrices obtained by the singular value decomposition of ***z*** with rank *r*, that is, $z\;=\;U\Sigma V^{T}$, and $S_{\beta }\left(\Sigma \right)$ is expressed as

(A15)
$$\begin{array}{ccc} & & S_{\beta }\left(\Sigma \right)\\ & & =\;\mathrm{diag}\left(\max\left(\sigma_{i}-\beta ,\;0\right)\right)\;\left(i\;=\;1,2,\cdots ,r\right), \end{array}$$

with $\sigma_{i}$ being the *i*th singular value.

In the case of LTV, α_1_ is replaced by α, and ***x*** and ***y*** are expressed as [***L*** ***S***]*^T^* and [***p q***]^*T*^, respectively. ***K*** is expressed as

(A16)
$$K=\left(\begin{array}{cc} A & A\\ 0 & \nabla_{3} \end{array}\right),$$

and the equations including ***r*** and ***v*** in Equation (A13) are neglected.
